# The halo sign as a chest computed tomography finding of COVID-19

**DOI:** 10.31744/einstein_journal/2020AI5742

**Published:** 2020-04-24

**Authors:** Lucas de Pádua Gomes de Farias, Helena Alves Costa Pereira, Eduardo Pinheiro Zarattini Anastacio, Fernanda Formagio Minenelli, Gustavo Borges da Silva Teles

**Affiliations:** 1 UnitedHealth Group São PauloSP Brazil UnitedHealth Group, São Paulo, SP, Brazil.; 2 Hospital Israelita Albert Einstein São PauloSP Brazil Hospital Israelita Albert Einstein, São Paulo, SP, Brazil.

**Figure 1:**
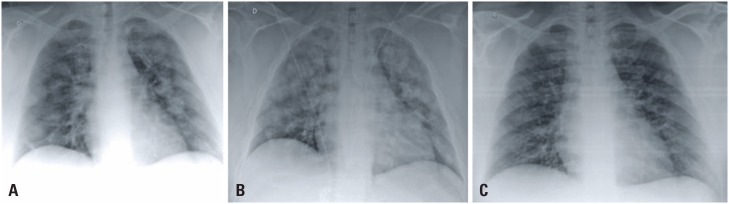
Bedside chest radiography at hospital admission (A), on day 2 (B) and day 10 (C) of hospitalization showed diffuse opacities in both lungs. Many of these opacities are seen to have nodular configuration that were more evident on the 2nd day of hospitalization (B)

**Figure 2:**
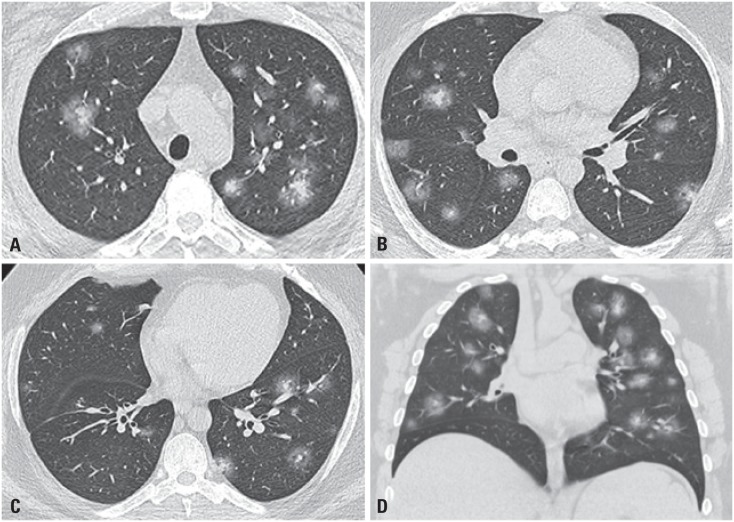
Halo sign. Axial (A and C) and coronal (D) images in multislice computed tomography show small multiple solid nodules with ground-glass halo in upper (A), middle (B), and lower (C) lung fields, representing the halo sign. Other nodular opacities with ground-glass attenuation can be seen

A 36-year-old male patient from New Jersey, USA, with obesity, hypertension and diabetes, was admitted to our service presenting excessive sweating, tachypnea, tachycardia, fever (38.6°C), and blood oxygen level of 90%. The patient reported that 5 days before he had sought medical care in the United States for dry cough, nasal congestion, malaise and fever. Chest radiography (Figure 1) and computed tomography (CT) (Figure 2) at admission showed multiple small nodular opacities and diffuse consolidations in both lungs, with central and peripheral distribution as well as with ground-glass halo, which corresponds to the halo sign.

The reverse transcription polymerase chain reaction (RT-PCR) assay for coronavirus identified the viral RNA in the patient-collected nasopharyngeal swab.

Computed tomography findings of pneumonia in patients with severe acute respiratory syndrome-related coronavirus-2 (SARS-CoV-2), the coronavirus diseases 2019 (COVID-19), are nonspecific and they can be found in other viral etiologies and in organizing pneumonia. The most common aspect includes multifocal ground-glass opacities associated or not with areas of consolidation or septal thickness (the “crazy-paving” pattern), and bilateral distribution involving mainly the peripheral lung regions.^(^^1^^)^ The CT can be used for symptomatic patients, including higher sensibility reports, but with limited specificity concerning the standard molecular test.^(^^2^^,^^3^^)^ However, this procedure is not recommended for screening by the majority of medical societies.^(^^1^^)^

The halo sign is not characteristic or a frequent presentation among patients with COVID-19 lung injury. There are few reports on this subject in published literature, although the halo sign has been observed in other viral pneumonias.^(^^4^^)^ Recently, two other cases reported the halo sign, a 27-year-old female^(^^5^^)^ and a 46-year-old male patients.^(^^6^^)^

This CT sign was first described in 1985 as geographic areas of low lung attenuation in the lung (ground-glass opacity) surrounding a nodule or mass, and it was associated with angioinvasive pulmonary aspergillosis in patients with acute leukemia.^(^^7^^)^ In these reported cases, the ground-class represents the perinodular alveolar hemorrhage caused by pulmonary infarction due to the angioinvasive aspergillosis.

So far, numerous other etiologies with this tomography presentation have been already described.^(^^4^^,^^8^^,^^9^^)^ In immunocompromised patients, in addition to angioinvasive aspergillosis (most frequent in neutropenic individuals), the possibility of Kaposi sarcoma should be also considered in HIV-positive patients with low CD4 T-cell counts. However, in immunocompromised patients, the halo sign can be found in primary adenocarcinoma of the lung (mainly when there is lepidic component), metastasis (specially associated with hemorrhagic component), and in other inflammatory and infectious conditions such as granulomatosis with polyangiitis, organizing pneumonia, some bacterial, fungi, and viral infections.^(^^4^^,^^8^^,^^9^^)^

Although the halo sign constitutes a CT finding with large differential diagnosis and it is rarely found in pneumonias caused by SARS-CoV-2, this CT sign in individuals under COVID-19 investigation can help in this disease diagnosis and management, particularly during the current pandemic.
