# Supplementary material for: The halo sign as a chest computed tomography finding of COVID-19
Source: Einstein (Sao Paulo). 2020 Apr 24;18:eAI5742. doi: 10.31744/einstein_journal/2020AI5742 (PMC7186001; doi:10.31744/einstein_journal/2020AI5742)
Supplement: Translation

# Translation: O sinal do halo como apresentação tomográfica pulmonar na COVID-19

**Authors:** Lucas de Pádua Gomes de Farias, Helena Alves Costa Pereira, Eduardo Pinheiro Zarattini Anastacio, Fernanda Formagio Minenelli, Gustavo Borges da Silva Teles

**Affiliations:** 1 São PauloSP Brasil UnitedHealth Group, São Paulo, SP, Brasil; 2 São PauloSP Brasil Hospital Israelita Albert Einstein, São Paulo, SP, Brasil

Paciente do sexo masculino, 36 anos de idade, natural e procedente de Nova Jersey (Estados Unidos), obeso, hipertenso e diabético, compareceu ao nosso serviço sudoreico, taquipneico, taquicárdico e febril (38,6°C) e saturando 90% em ar ambiente. Relatou que, há 5 dias, procurara um serviço médico nos Estados Unidos por queixa de tosse seca, coriza, mal-estar e febre referida. Na admissão, o paciente realizou radiografia (Figura 1) e tomografia computadorizada (Figura 2) de tórax, que evidenciaram múltiplas pequenas opacidades nodulares e consolidações esparsas por ambos os pulmões, com distribuição periférica e central, acompanhadas de halo em vidro fosco, configurando o sinal do halo. A pesquisa da infecção por coronavírus por meio de reação em cadeia da polimerase com transcrição reversa (RT-PCR) detectou a presença do RNA do vírus no material colhido da nasofaringe do paciente.

**Figura 1:** Radiografias de tórax realizadas no leito no dia da admissão hospitalar (A), no segundo (B) e no décimo dias de internação (C) evidenciam opacidades difusas em ambos os pulmões, muitas delas com configurações nodulares, mais evidentes no segundo dia da internação hospitalar (B)

**Figura 2:** Sinal do halo. Imagens axiais (A a C) e coronal (D) de tomografia computadorizada multidetectores evidenciam múltiplos pequenos nódulos sólidos com halo em vidro fosco nos campos pulmonares superiores (A), médios (B) e inferiores (C), caracterizando o sinal do halo. Note as demais opacidades nodulares com atenuação em vidro fosco

Os achados tomográficos da pneumonia na doença pelo novo coronavírus tipo 2 (SARS-CoV-2), a *coronavirus disease 2019* (COVID-19), são inespecíficos e podem ser encontrados em outras etiologias virais e nas pneumonias em organização. O aspecto mais comum inclui opacidades em vidro fosco pulmonares multifocais, associadas ou não às áreas de consolidação ou espessamento septal (configurando aspecto de “pavimentação em mosaico”), apresentando distribuição bilateral, principalmente nas regiões pulmonares periféricas.^(^^1^^)^ A tomografia computadorizada pode ser usada nos pacientes sintomáticos, inclusive com relato de maior sensibilidade, mas especificidade limitada em relação ao teste molecular padrão,^(^^2^^,^^3^^)^ porém não é recomendada para o rastreio pela maioria das sociedades.^(^^1^^)^

O sinal do halo não constitui apresentação característica ou frequente nos pacientes com comprometimento pulmonar na COVID-19, com pouquíssimos relatos na literatura, apesar de sua possível apresentação em algumas outras pneumonias virais.^(^^4^^)^ Foram observados outros dois casos recentemente relatados – uma paciente do sexo feminino, de 27 anos de idade,^(^^5^^)^ e outro do sexo masculino, com 46 anos de idade.^(^^6^^)^

Identificado por tomografia computadorizada, o sinal do halo foi descrito inicialmente em 1985 como uma zona de baixa atenuação (em vidro fosco), em forma de halo, circundando uma massa ou nódulo, e representava as lesões pulmonares por aspergilose angioinvasiva em pacientes com leucemia aguda.^(^^7^^)^ Nesses casos, o halo em vidro fosco representa hemorragia alveolar perinodular como consequência do infarto pulmonar provocado pela angioinvasão do *Aspergillus*.

Até o presente momento, diversas outras etiologias com esta apresentação tomográfica já foram descritas.^(^^4^^,^^8^^,^^9^^)^ Em pacientes imuncomprometidos, além da aspergilose angioinvasiva (mais frequente em indivíduos neutropênicos), a possibilidade de sarcoma de Kaposi também pode ser considerada nos pacientes com sorologia positiva para o HIV e baixa contagem de linfócitos T CD4. Já nos pacientes imunocompetentes, o sinal do halo pode ser encontrado no adenocarcinoma primário de pulmão (principalmente quando existe componente lepídico), nas metástases (especialmente com componente hemorrágico associado) e em outras condições inflamatórias e infecciosas (granulomatose com poliangeíte, pneumonia em organização, algumas infecções bacterianas, fúngicas e virais).^(^^4^^,^^8^^,^^9^^)^

Embora o sinal do halo seja um achado tomográfico com amplo diagnóstico diferencial e tenha apresentação rara nas pneumonias causadas pelo SARS-CoV-2, seu reconhecimento nos indivíduos com quadro clínico sugestivo de COVID-19 pode ajudar no diagnóstico e no manejo destes pacientes, principalmente no atual cenário de pandemia.
